# Assessment of Workplace Social Encounters: Social Profiles, Burnout, and Engagement

**DOI:** 10.3390/ijerph18073533

**Published:** 2021-03-29

**Authors:** Michael P. Leiter

**Affiliations:** School of Psychology, Deakin University, Geelong, VIC 3220, Australia; Michael.leiter@acadiau.ca

**Keywords:** workplace civility, workplace incivility, burnout, engagement, intimidation

## Abstract

Employed people (*N* = 826) completed questionnaires including the Social Encounters Scale that assessed civility, incivility, and intimidation from supervisors, coworkers, and respondents on identical frequency scale. Factor analyses, correlations, and profile analysis addressed the first research question by demonstrating the benefits of assessing various dimensions of workplace social dynamics on a common rating scale. A subsample (*N* = 275 completed a second survey, confirming consistency over time. To address the second research question a Latent Profile Analysis (LPA) identified five social profiles: Civil, Low Contact, Uncivil Coworkers, Uncivil Supervisor, and Uncivil. These profiles were associated with distinct perceptions of the work environment, addressing the third research question. To address the fourth research question, crosstabulation with a profile structure based on the Maslach Burnout Scale demonstrated close links of workplace social culture with psychological connections with work.

## 1. Introduction

Jobs are relationships of people with worksettings, the parameters of which are defined by both formal and informal contracts. These connections of people with institutions encompass varieties of relationships among people that they realize through social encounters that occur throughout workdays. These encounters may be brief or extended, shallow or deep, rewarding or distressing. In light of the importance of belonging as a primary motive [1], these encounters have a role in shaping how people experience their worksetting. It is not surprising that for the most part people get along well at work [2,3], given the importance of belonging and the capacity of people to solve problems when they arise. However, when disrespectful encounters thwart the motive to belong, there is a great potential for distress.

Persistently poor workplace cultures create problems for institutions, their leaders, and the people in affected workgroups. On the institutional level, laws in many jurisdictions make organizations liable for mistreatment occurring among employees [4]. In healthcare or education, poor workplace cultures can hurt an institutions accreditation standing [5]. For people, workplace incivility, even when of low intensity and unintended, is associated with distress. It is difficult to identify benefits of workplace mistreatment, but it persists nonetheless. As the problem seems unlikely to go away on its own, it is important that work psychology develop a precise understanding of its dynamics.

### Social Encounters Scale

The Social Encounters Scale provides a diverse and nuanced perspective on employees’ social encounters at work. By assessing civility, incivility, and intimidation, both instigated and received, the measure lends itself to person-oriented analysis that captures distinct patterns of involvement in workplace social environments.

Many research studies have measured incivility as the frequency of incidents, using the Workplace Incivility Scale [6] as well as the Straightforward Incivility Scale (Leiter & Day, 2012). However, studies have often measured workplace civility as a general assessment of workplace social climate or culture, asking respondents to indicate their degree of agreement or disagreement with generally descriptive items. For example, the CREW Civility Scale [7] asks how strongly people agree or disagree with the statement, “People treat each other with respect in my work group”.

The Social Encounters Scale assesses both civility and incivility as well as intimidation on the same seven-point frequency scale from Never (0) to Every Day (6). The measure assesses received encounters from supervisors and coworkers. It also assesses the same items as instigated civility, incivility, and intimidation from respondents towards others at work. The intention was to develop an integrated assessment of individuals’ experience of social encounters. The structure of the scale is intended to capture qualitative distinctions between encounters with coworkers and those with supervisors. That is, the scale implicitly proposes that encounters with supervisors differ from those with coworkers because of the authority issues inherent in supervisory relationships in contrast to the social expectations among colleagues. Further, people consider their instigated social behavior differently from receive social behavior. They understand their own intentions to act respectfully or incivility differently than they understand the intentions of other people. Although recognizing that respondents’ view of their instigated social behavior differs qualitatively from their views of received social encounters, the SES operationalizes them with parallel items.

The contribution of this article is to present a measure of workplace social encounters that builds on previous measures of workplace incivility. Second, the article demonstrates the utility of person-centered analysis to elucidate responses to the survey. Person-centered analysis has a distinct relevance to the constructs under consideration in that social behavior is highly contextualized. People experience their own social behavior in the context of the social behavior that they receive from others. Further, people interpret the negative social encounters they receive, such as incivility, in the context of the positive social encounters they receive in the same setting. As such, analyses that integrate positive and negatively valenced behaviors from multiple sources into a profile may have greater resonance with respondents’ experience than measures of each taken in isolation. As such, this approach may provide valuable insights into the impact of interventions designed to improve the social context of work.

Latent Profile Analysis (LPA) examines person-by-person patterns of relationships among groups of constructs. When evaluating a group of highly inter-correlated constructs, the results evolve into high, medium, and low on all constructs, adding little to what can be gleaned from examining the constructs separately. However, when examining groups of constructs with diverse sets of inter-correlations, LPA identifies distinct patterns of scores that subsets of participants share that differ in meaningful ways from patterns exhibited by other subsets of participants. As such, person-oriented analysis can highlight important subgroups within a data set who depart in meaningful ways from the overall correlations across a sample. For example, supervisor civility is generally correlated with coworker civility with positive associations with workplace measures. However, a minority of respondents may have frequent civil encounters with coworkers but not with their supervisors. This profile may have patterns of associations with workplace measures that are distinct from those of colleagues with frequent civility from both coworkers and supervisors. The capacity to distinguish these profiles may provide valuable for designing workplace interventions.

A profile framework for workgroup social environments would provide an opportunity for a fresh examination of the relationship of civility and incivility with job burnout. Leiter and Maslach [8] confirmed a five profile that distinguished Engaged (low exhaustion, low cynicism, high efficacy) and burnout (high exhaustion, high cynicism, low efficacy) from three profiles in which only one of the three Maslach Burnout Inventory (MBI) subscales had problematic scores: Ineffective (low exhaustion, low cynicism, low efficacy), Overextended (high exhaustion, low cynicism, high efficacy), and Disengaged (low exhaustion, high cynicism, high efficacy). Research has established that civility and incivility are associated with job burnout [9,10]. Relating the MBI-based Worklife profiles with profiles based on the SES would supplement these findings with person-centered perspectives on employees’ distress at work.

Job burnout is a syndrome of exhaustion, cynicism, and inefficacy that reflects a workplace stress that has not been successfully managed [11]. Its definition differs from chronic exhaustion by the inclusion of cynicism and inefficacy. While not a disease, burnout is a major life crisis that is associated with mental and physical distress. The syndrome reflects a breakdown in employees’ relationships with work that includes but goes beyond unmanageable work demands to include serious mismatches regarding community, values, fairness, and [12].

The first research questions in this exploratory analysis is does the SES have a sound factor structure despite the parallel wording of items across the subscales? That is, does designating the source of social behavior—supervisor, coworker, or self—distinguish respondents’ experience sufficiently that these items cluster in their designated subscales?

The second research question concerns the person-focused analytic approach: do the subscales cluster to reflect distinct profiles of score that are add information to what could be discerned through variable focused analyses?

The third research question concerns convergent validity. Does the SES scale correspond with a measure of psychological safety in a manner consistent with the measure with low psychological safety being associated with uncivil profiles and high psychological safety associated with civil profiles? Additionally, does the SES scale correspond with measures of supervisor and coworker trust consistent with the definition of profiles in terms of incivility sources? That is, does a profile defined by coworker incivility have distinctly low levels of coworker trust and a profile defined by supervisor incivility have distinctly low levels of supervisor trust.

The fourth research question concerns the correlations of the profiles with the dimensions of job burnout: are the profiles associated with the three dimensions of job burnout such that greater incivility is associated with a greater prevalence of distressed worklife profiles. Previous research has established that burnout is closely related to workplace incivility using regression-based analyses [9,10]. This analysis will supplement those findings by considering the experiences of people with distinct profiles on both the dimensions of burnout and the variations in social encounters.

## 2. Method

### 2.1. Participants

Participants (*N* = 826) provided complete responses to surveys that assessed social encounters and perceptions of their workplaces. The sample included employed people in Australia from various industries including hospital-based healthcare providers (321), Service Professionals (242), police officers (107), and other (*N* = 156). The average age was 36.43 years (SD = 12.26); there were 261 males, 558 females, 6 other, and 1 not responding. Of the 1810 invitations to participant, 826 were completed the first survey (45.63%). A subset of these respondents (*N* = 275) completed a second survey administered at a six-month interval. This sample included 106 males, 167 females, and 2 other; the sample had an average age of 36.29 (SD = 12.00).

The sample was gathered through on-line workplace surveys as part of a research project on workplace civility. Invitations to participate were sent to lists of employees through the human resources department of participating organizations. The inclusion criterion was full or part time employment. Only people meeting this criterion were included in the study.

### 2.2. Procedure

All surveys occurred as part of research projects examining workplace civility and its relationships management practices and job burnout. All survey were administered online. After obtaining ethics approval (HREC/17/WH/175) and authorization from management, participants received invitations to complete follow a link to the survey site. Participants were free to withdraw participation at any time.

### 2.3. Measures

The Social Encounters Scale (SES) [13] produces nine subscales based on 23 items: Supervisor Civility, Supervisor Incivility, Supervisor Intimidation, Coworker Civility, Coworker Incivility, Coworker Intimidation, Instigated Civility, Instigated Incivility, and Instigated Intimidation.

The SES supervisor sections begin with, “Over the past month, how often has your own supervisor behaved in the following ways?” The coworker sections refers instead to coworkers. The instigated section begins asking respondents how often they acted in this way towards others, with the items reworded as needed.

- A sample items for civility: “Was appreciative of others and their work.”
- A sample item for incivility: “Behaved rudely to you (e.g., gestures, facial expressions, etc.).”
- A sample item for intimidation: “Intentionally threatened you.”

Respondents rated the items on a seven-point frequency scale: Never (0), Sporadically (1), Now and Then, (2), Regularly (3), Often (4), Very Often (5), Daily (6).

Trust in supervisors and trust in coworkers were measured by six items from Cook and Wall’s Interpersonal Trust at Work Scale [14]. This scale measures two aspects of trust (1) faith in intentions and (2) confidence in competence. Items are averaged to obtain scores ranging from 1 = strongly disagree, 5 = strongly agree. In the current study, the internal reliability was α = 0.82 for supervisor trust and α = 0.87 for coworker trust.

Psychological Safety. Three items from a scale developed by Edmondson [15] measured psychological safety. Participants used a 5-point Likert-type scale (ranging from 1-strongly disagree to 5-strongly agree) to indicate the extent to which the felt safe in their group (e.g., “It is safe to take a risk in this work group.”). Cronbach’s alpha was ƒÁ = 0.81.

Burnout was measured with the Maslach Burnout Inventory-General Scale (MBI-GS) [16] that produces three subscales: exhaustion, cynicism, and efficacy. Respondents rate items on a 7-point frequency scale form 0 (never) to 6 (every day).

The analysis reported here use the five profiles defined by Leiter and Maslach [8], based on Latent Profile Analysis of the MBI-GS: Engaged (positive on all three subscales: low exhaustion, low cynicism, high efficacy), Ineffective (negative only on efficacy: low exhaustion, low cynicism, low efficacy), Overextended (negative only on exhaustion: high exhaustion, low cynicism, high efficacy), Disengaged (negative only on cynicism: low exhaustion, high cynicism, high efficacy), and Burnout (negative on all three subscales: high exhaustion, high cynicism, Low efficacy). For this analysis the MBI scores for participants were combined with a normative database of 45,000 respondents on the MBI-GS from 1998 to 2016 that was the source of normative data on the MBI-GS [16]. Including a sample’s data into a larger normative data set assigned cases in the sample to profiles established in the larger context. For example, a case in the sample would be assigned to Overextended only if the exhaustion score was negative relative to the norms. A LPA on the sample alone would assign a case to Overextended if exhaustion was negative relative to that sample alone.

This study operationalizes engagement as low exhaustion, low cynicism, and high efficacy as measured on the MBI. This approach combines both positively and negatively worded items to define engagement as a state of being energetic, involved, and efficacious as defined by Maslach and Leiter [17].

## 3. Results

### 3.1. Confirmatory Factor Analysis

To address the first research question we conducted a confirmatory factor analysis (CFA) of Social Encounters Scale (SES) using EQS (EQuatonS) (Multivariate Software, Inc, Temple City, CA, USA) [18] on 826 cases for which there were complete data. The analysis proceeded with nine variables: Supervisor Civility, Supervisor Incivility, Supervisor Intimidation, Coworker Civility, Coworker Incivility, Coworker Intimidation, Instigated Civility, Instigated Incivility, and Instigated Intimidation.

The factors were freed to correlate; parallel error covariances were freed: e. g., Supervisor Civility item 1 error with Coworker Civility item 1 error and with Instigated Civility item 1 error. In light of the skewed distribution of many items, the analysis used the robust option that accommodates for departures from normality. The indicators of fit, including chi-square, comparative fit index, and root mean square error of approximation (RSEA) all indicated an excellent fit (ƒÖ^2^_(159)_ = 338.35, *p* < 0.001, CFI = 0.949; RMSEA = 0.043 (90% Confidence Interval: 0.036 to 0.049)).

### 3.2. Relationships among Measures

Table 1 displays the descriptive statistics and correlations among the SES subscales with the exhaustion, cynicism, and efficacy from the Maslach Burnout Inventory. In general civility has moderate negative correlations with incivility and intimidation that have positive correlations with one another. Exhaustion and cynicism have positive correlations with incivility and intimidation; efficacy has positive correlations with civility.

Instigated civility is positively correlated with supervisor civility (*r* = 0.42), but more strongly correlated with coworker civility (*r* = 0.59; t = 5.88, *p* < 0.001). Instigated incivility is correlated with supervisor incivility (*r* = 0.27), but more strongly positively correlated with coworker incivility (*r* = 0.42; t = 4.45, *p* < 0.001).

To assess consistency over time, we calculated correlations of scores over a six month interval for 275 participants who provided complete data at both assessments. As indicated in Table 2, measures of civility and incivility showed a moderate level of stability with correlations from 0.44 to 0.67. Intimidation was less consistent (supervisor intimidation; *r* = 0.39; coworker intimidation; *r* = 0.28; instigated intimidation; *r* = −0.01). As indicated in Table 1, intimidation occurred with less frequency than the other two forms of social encounters.

### 3.3. SES Profiles

To address the second research question we used a series of Latent Profile Analyses (LPA) using Mplus8 [19] evaluated models with various numbers of profiles. LPA uses an interactive process, the results of which provide indicators of model fit, none of which are considered definitive on its own. In combination they can inform decisions on selecting the number of profiles to pursue. The indicators include the Akaike information criterion (AIC), the Bayesian information criterion (BIC), and the Lo-Mendell Rubin adjusted likelihood ratio. For AIC and BIC, smaller values indicate a better fit [20]; for Lo-Mendell and the Bootstrapped Likelihood Ratio Test, a significant value indicates a better fit than provided by a model with one less profile [21]. Entropy indicates the probability of accurate assignment of cases to a profile. In addition to these statistical indicators, one examines the extent to which the resulting profiles make sense in light of the theoretical underpinnings of the approach. Finally, profile solutions with very small numbers of participants in any of the profiles are not viable.

We conducted the LPA on four indicators from the SES:

Supervisor Civility,

Supervisor Incivility,

Coworker Civility,

Coworker Incivility.

We limited the SES indicators to these four constructs because they provided sufficient complexity by including both positive and negative encounters from two sources. We omitted the Intimidation constructs because they were highly skewed, with a large majority of responses as zero. These distributions violate the LPA analysis assumptions and would produce invalid outcomes or fail to converge. We omitted the Instigated constructs because we intended to examine the extent to which profiles based on received encounters predicted instigated social behavior. Received and instigated social behavior have qualitatively different statuses for respondents, such that restricting the LPA to received behavior provides a more valid perspective on a workgroup’s social dynamics. Although a model including both received and instigated social behavior is of interest, it would be appropriate for addressing research questions other than those considered in this study.

The results of the LPA produced a five profile solution (See Table 3). The fit indicators did not provide definitive guidance regarding the selection of the model. The fit indices show AIC and BIC decreasing with additional profiles. Entropy changed very little, remaining in an acceptable range throughout the range of models tested. The BLRT level remained consistent throughout. The five profile solution was chosen the improvement of indices over the four profile solution and because the smallest profile N was larger than that for the six profile solution. The six profile solution had two profiles that were smaller than that for the five profile solution. The five profiles are described in Table 4.

- The Civility profile includes frequent civility from both sources and infrequent incivility from both sources.
- The Incivility profile at the opposite pole has infrequent civility from both sources and frequent incivility from both sources.
- Low Contact has relatively infrequent encounters on all measures.
- Uncivil Coworker has infrequent coworker civility and frequent coworker incivility with moderate encounters with supervisors.
- Uncivil Supervisor has infrequent supervisor civility and frequent supervisor incivility with moderate encounters with coworkers.

**Figure 1:**
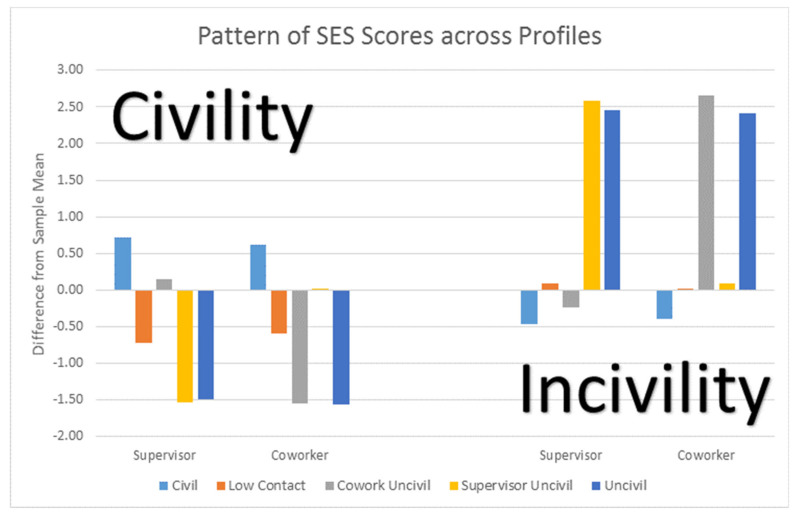
Pattern of SES scores across profiles.

Figure 1 the pattern of scores for Supervisor and Coworker Civility and Incivility across the five profiles. The Civil profile has both supervisor and coworker civility above the mean with supervisor and coworker incivility below the mean. The Incivility profile has the opposite pattern. Low contact has civility from both sources below the mean with incivility from both sources at the mean. Note that incivility occurs on average infrequently (i.e., the means for both supervisor and coworker incivility are less than 0.80 while the means for the respective civility scores are above 3.00 on a 0 to 6 scale). The Coworker Incivility profile combines average levels of supervisor civility with low levels of coworker civility. It also has average levels of supervisor incivility with high levels of coworker incivility. The Supervisor Incivility profile has the opposite pattern of moderate scores for coworker encounters and negative scores for supervisor encounters.

**Figure 2:**
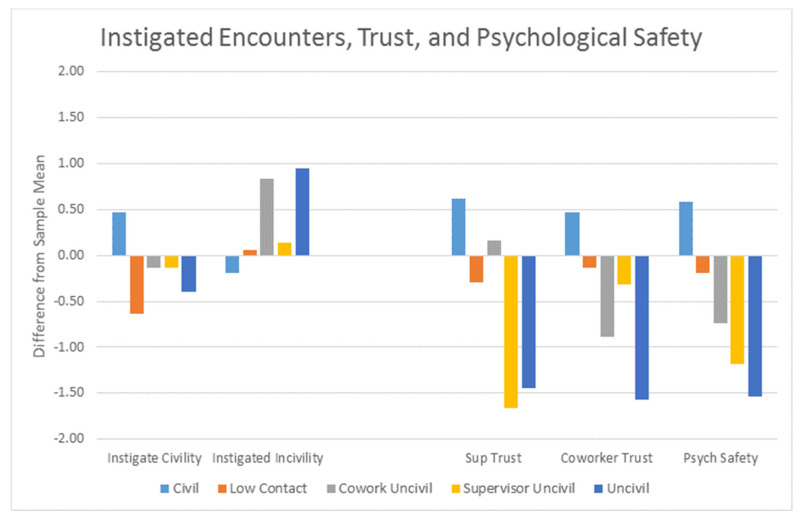
Instigated encounters, trust, and psychological safety.

To address the third research question Figure 2 displays the scores for instigated encounters, trust in supervisors and in coworkers, and psychological safety across the profiles. Tukey tests (*p* < 0.05) within an ANOVA found the Civil profile to score higher on instigated civility (M = 4.70) than any of the other profiles. For Instigated incivility, both the Civil (M = 0.25) and Low Contact (M = 0.39) profiles score lower than the other three profiles. The trust contrasts indicate specific patterns for supervisor versus coworker encounters. For supervisor trust, the lowest level was for Uncivil (M = 1.98) that did not differ from Supervisor Uncivil (M = 1.74); both were lower than the supervisor trust level for Low Contact (M = 3.25) and Coworker Uncivil (M = 3.76). Civil (M = 4.27) had the highest level. Uncivil also had the lowest level of coworker trust (M = 2.50) followed by Coworker Uncivil (M = 3.11) that in turn was lower than Supervisor Uncivil (3.61) and Low Contact (M = 3.76). Civil (M = 4.28) had the highest level. For psychological safety the lowest level was for Uncivil (M = 1.55) that was lower than Supervisor Uncivil (M = 1.91) and Coworker Uncivil (M = 2.37) that in turn were lower than the psychological safety level for Low Contact (M = 2.92). Civil (M = 3.70) had the highest level.

**Figure 3:**
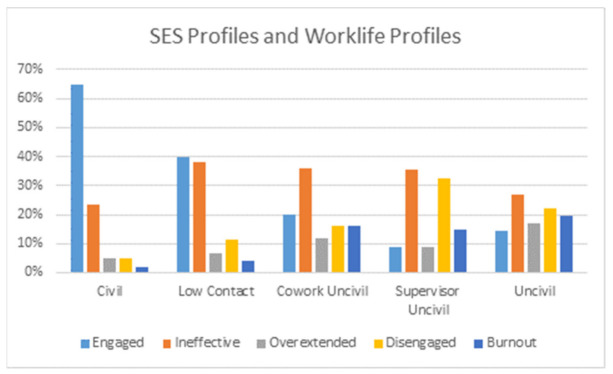
SES profiles and worklife profiles.

To address the fourth research question Figure 3 and Table 5 display the cross-tabulation of SES profiles with Worklife Profiles based on the three subscales of the Maslach Burnout Inventory. The two profiles were related to one another (ƒÖ^2^_(16)_ = 153.85, *p* < 0.001). As indicated in Figure 3, the Engaged worklife profile (65%) is over-represented in the Civil SES Profile, followed by Ineffective, with few of the distressed profiles (Overextended, Disengaged, and Burnout). Low Contact also has few of the distressed worklife profiles, but has a nearly equal proportion of Engaged and Ineffective worklife profiles. For the Coworker Uncivil SES profile, the distressed MBI profiles are more prevalent while Engaged and Ineffective make up a smaller proportion. Supervisor Uncivil has few Engaged respondents and a larger proportion of Overextended and Burnout respondents. The Uncivil SES profile is overrepresented with all three distressed profiles.

## 4. Discussion

A person-focused approach to analyzing the Social Encounters Scale demonstrated that the measure provides a nuanced perspective on employees’ social dynamics. By providing distinct measures of civil and uncivil encounters from supervisors and coworkers as well as the instigated encounters from respondents, the measure provides the basis for complex and informative profiles of respondents’ social dynamics at work. The confirmatory factor analysis supported the SES’s structure that distinguished three types of social encounters (civil, uncivil, and intimidation) from three sources (supervisor, coworkers, and self), addressing the first research question. The latent profile analysis distinguished five profiles: Civil, Low Contact, Coworker Uncivil, Supervisor Uncivil, and Uncivil, addressing the second research question presented in the introduction. Contrasts among the profiles on trust and psychological safety suggest that applying person-centered analyses to the Social Encounters Scale can provide distinct and useful perspectives on workplace civility, addressing the third research question. The alignment of the SES profiles with the profiles based on the Maslach Burnout Inventory identify social dynamics associated with burnout and related states, addressing the fourth research question. The analyses confirmed the study hypotheses regarding the structure of the measures and their functionality in person-oriented analyses. The profile framework defines engagement as the opposite of burnout [17]. Other definitions of engagement do not position it as directly opposite to burnout, although those measures are consistently correlated negatively with burnout measures [22]. The potential for improved workplace civility as a means of improving employees’ psychological connections with their work are explored.

### 4.1. Factor Structure

The CFA confirmed that the SES differentiates ratings of three types of social encounters (civility, incivility, and intimidation) from three sources (supervisor, coworkers, and self). These differentiations permit assessment of a variety of social encounters on the same frequency scale, capturing some of the diversity in respondents’ social encounters throughout their workdays. The range of scales indicate that across all three sources, civility occurs much more frequently than incivility that in turn occurs more frequently than intimidation. The parallel structure of the measure can in this way provide a metric for improving civility while also tracking decreases in incivility and intimidation during intervention programs. The parallel assessment of instigated social encounters beside received social encounters may also encourage people viewing themselves as active participants rather than passive observers in their workplace communities. The distinction of coworker from supervisor encounters—both civil and uncivil—supports considering those relationships has having qualitatively different implications for employees.

The correlations of intimidation with incivility from the same source supports the idea that low intensity incivility shares cultural qualities with more intense forms of social mistreatment. The strong correlation of supervisor with coworker intimidation is also consistent with intimidation as a workplace cultural issue.

The capacity of the SES to make parallel assessments of civility and incivility, both instigate and received, makes a contribution to a burgeoning research field [9]. Workplace social behavior have become a focus of increasing concern and theoretical interest over the past decade and appears to have considerable momentum for continuing in that trajectory. The complexity of assessing social environments call for a measure that can address multiple facets of social encounters, but do so in an integrated fashion. The research work to date has established causes and consequences of workplace incivility [23], but it needs a more solid foundation to further intervention studies designed to improve workplace civility. The measures and constructs upon which researchers are using to assess change need precise focus and internal consistency to further intervention studies [24].

In structural modelling, there is potential to use instigated incivility as an outcome variable with received incivility as predictors. The focus on instigated incivility demonstrate the role of reciprocity in perpetuating problematic social environments within workgroups [25,26]. It is important that measures of received and instigate incivility have consistent structures to permit through examination of their interdependencies.

### 4.2. Profiles

The Latent Profile Analysis (LPA) favored a consistent five-profile structure. The Civil and Uncivil profiles established a contrast with scores all positive (high civility with low incivility) versus all negative (low civility with high incivility), respectively. In addition, the analysis identified additional profiles that departed from the usual correlation format: Low Contact with relatively infrequent encounters on all forms of encounter, Uncivil Coworker with negative encounters only with coworkers and Uncivil Supervisor with negative encounters only with supervisors. These profiles capture five distinct patterns of social encounters within workgroup cultures. Although it recognizes situations that are fully negative in the Uncivil profile or fully positive in the Civil profile, the framework accommodates mixed situations with Coworker Uncivil and Supervisor Uncivil profiles. Completing the framework is the Low Contact profile in which few social encounters of any kind occur.

The relationships of SES profiles with the Worklife Profiles based on the MBI demonstrates a capacity to identify subgroups within larger organizations who would benefit from additional support or structured interventions to alleviate distress. For example, only two percent of those in civil social environments were in the Burnout profile, but 15 to 20 percent of those in uncivil social environments (coworker uncivil, supervisor uncivil, and uncivil) were in the Burnout profile. This information suggests that resources would be best devoted to focusing efforts on areas with civility challenges. The primary difference between the Engaged and Ineffective profiles was the larger number of civil social encounters reported by people in the Engaged profile. This pattern supports the proposition that positive social encounters play a definitive role in sustaining an engaged approach to work. This analysis provides additional information to research that has established links between burnout in workplace incivility [27,28].

### 4.3. Limitations

Survey measures of workplace social encounters share shortcomings with those of other constructs in that they rely on respondents’ self-reports of their recollections. A problem evident when assessing behavior is a self-serving bias to present oneself in a good light. The sample size was limited by restricting the analysis to a longitudinal sample, resulting in profiles that included less than 5% of the sample. Further research is needed to apply the analysis to larger samples.

The LPA analyses did not include intimidation because of its highly skewed distribution, dominated by zero entries. While it is encouraging that intimidation occurs rarely, it remains a critical challenge to workplace communities.

An overall limitation is that the data for the study is derived from self-report questionnaires. Further research examining the alignment of questionnaire responses with observations and with qualitative data will inform the scales’ validity.

### 4.4. Implications for Practice

The Social Encounters Scale provides a practical means of assessing diverse information on social encounters among members of workgroups. The consistent assessment of civility and incivility makes a practical in interventions designed to reduce workplace mistreatment often contain a mechanisms designed to increase civility in the first instance [2,3]. The scale thereby provides an assessment of the program objective of reducing incivility while providing a measure of the immediate mechanism’s action to increase civility. Secondly, contrasting received with instigated scores indicates the extent to which participants share responsibility for their workgroup culture in contrast to experiencing their culture as imposed by other people. These qualities can help to support much-needed research on workplace interventions to improve workplace cultures and to reduce mistreatment.

### 4.5. Future Directions

The profile approach can be additionally useful when determined on a workgroup level. Workgroup profiles would emphasize the shared culture of their workgroup. This approach can distinguish groups for which civility is intrinsic to their work from those that struggle with incivility or social contact. Groups may vary in the extent to which members share a consistent view of their culture. That is, civility may be a consistent experience for only a subset of group members while others participate in more strained social encounters. The capacity to capture the complexity and diversity of workplace social encounters is key to refining theory and evaluating interventions in this important dimension of organizational psychology.

## Figures and Tables

**Table 1:** Means, standard deviations, alphas, and Correlations among variables at Time 1.

Measure	Mean	SD	α	2	3	4	5	6	7	8	9	10	11	12
1 Exhaustion	2.16	1.27	0.88	0.62	−0.10	−0.25	0.37	0.22	−0.21	0.30	0.16	−0.04	0.18	0.02
2 Cynicism	1.76	1.35		0.86	−0.27	−0.31	0.39	0.25	−0.25	0.30	0.18	−0.13	0.20	0.00
3 Efficacy	3.96	1.07			0.78	0.21	−0.03	0.08	0.28	−0.08	0.04	0.34	−0.05	0.03
4 Supervisor Civility	3.65	1.52				0.91	−0.59	−0.31	0.56	−0.33	−0.22	0.42	−0.15	−0.03
5 Supervisor Incivility	0.69	1.06					0.85	0.59	−0.34	0.52	0.34	−0.13	0.27	0.11
6 Supervisor Intimidation	0.18	0.69						0.90	−0.22	0.36	0.54	−0.11	0.18	0.18
7 Coworker Civility	3.82	1.41							0.91	−0.51	−0.26	0.59	−0.18	−0.05
8 Coworker Incivility	0.67	0.95								0.86	0.52	−0.15	0.42	0.12
9 Coworker Intimidation	0.16	0.56									0.85	0.01	0.30	0.24
10 Instigated Civility	4.44	1.09										0.84	−0.18	−0.11
11 Instigated Incivility	0.35	0.56											0.67	0.29
12 Instigated Intimidation	0.03	0.29												n/a

Note: *N* = 826; *r* = ±0.09, *p* = 0.01; *r* = ±0.07, *p* = 0.05; Cronbach’s alpha on main diagonal.

**Table 2:** Correlation of Time 1 with Time 2 SES constructs.

Subscale	Correlation
Supervisor Civility	0.50
Supervisor Incivility	0.44
Supervisor Intimidation	0.39
Coworker Civility	0.56
Coworker Incivility	0.44
Coworker Intimidation	0.28
Instigated Civility	0.67
Instigated Incivility	0.57
Instigated Intimidation	−0.01

Note: *N* = 275. Social Encounters Scale—SES.

**Table 3:** Cross-sectional Latent Profile Analysis.

Model	AIC	SSA-BIC	LMR *p*	BLRT *p*	Entropy	Smallest Profile *N*
Four	10,859.81	10,898.29	0.02	0.001	0.86	29 (3%)
Five	10,643.24	10,690.09	0.62	0.001	0.88	28 (3%)
Six	10,426.62	10,481.84	0.04	0.001	0.86	22 (2%)
Seven	10,339.67	10,403.25	0.44	0.001	0.86	12 (1%)

Note: *N* = 826, AIC Akaike Information Criterion, SSA-BIC Sample Size Adjusted BIC, LM*R* = Lo-Mendell Rubin adjusted Likelihood Ratio Test, BLRT Bootstrapped Likelihood Ratio Test.

**Table 4:** Profile patterns.

Profile	Supervisor Civility	Coworker Civility	Supervisor Incivility	Coworker Incivility	Total
Civil	High	High	Low	Low	464
Low Contact	Low	Low	Low	Low	334
Uncivil Coworker	Medium	Low	Medium	High	28
Uncivil Supervisor	Low	High	High	Low	42
Uncivil	Low	Low	High	High	45

**Table 5:** Relationship of social profiles with worklife profiles.

Profile	Civil	Low Contact	Coworker Uncivil	Supervisor Uncivil	Uncivil	Total
Engaged	65%	40%	20%	9%	15%	50%
Ineffective	24%	38%	36%	35%	27%	30%
Overextended	5%	7%	12%	9%	17%	7%
Disengaged	5%	11%	16%	32%	22%	9%
Burnout	2%	4%	16%	15%	20%	5%
Total	53%	35%	3%	4%	5%	100%

Note: *N* = 826; ƒÖ^2^_(16)_ = 153.85, *p* < 0.001.

## Data Availability

The data presented in this study are available on request from the corresponding author. The data are not publicly available due to requirements of ethics approval.
